# Immunotherapy Resumption/Rechallenge in Melanoma Patients after Toxicity: Do We Have Another Chance?

**DOI:** 10.3390/pharmaceutics15030823

**Published:** 2023-03-02

**Authors:** Sofia España Fernandez, Chen Sun, Carme Solé-Blanch, Aram Boada, Anna Martínez-Cardús, José Luis Manzano

**Affiliations:** 1 Medical Oncology Department, Catalan Institute of Oncology Badalona, 08916 Badalona, Spain; 2 Badalona-Applied Research Group in Oncology (B-ARGO), IGTP (Health Research Institute Germans Trias i Pujol), 08916 Badalona, Spain; 3 Department of Tumor Radiotherapy, The Second Affiliated Hospital of Zhengzhou University, Zhengzhou 450014, China; 4 Dermatology Department, Hospital Universitari Germans Trias i Pujol, 08916 Badalona, Spain

**Keywords:** immunotherapy, immune checkpoints, rechallenge, resumption, toxicity

## Abstract

Introduction: Immune checkpoint inhibitors (ICIs) have radically changed the prognosis of several neoplasias, among them metastatic melanoma. In the past decade, some of these new drugs have appeared together with a new toxicity spectrum previously unknown to clinicians, until now. A common situation in daily practice is that a patient experiences toxicity due to this type of drug and we need to resume or rechallenge treatment after resolving the adverse event. Methods: A PubMed literature review was carried out. Results: The published data regarding the resumption or rechallenge of ICI treatment in melanoma patients is scarce and heterogeneous. Depending on the study reviewed, the recurrence incidence of grade 3–4 immune-related adverse events (irAEs) ranged from 18% to 82%. Conclusion: It is possible to resume or rechallenge, but each patient should be evaluated by a multidisciplinary team for close monitoring and assessment of the risk/benefit ratio before initiating treatment.

## 1. Introduction

Immune checkpoint inhibitors (ICIs) have changed the treatment paradigm of several neoplasias. Currently, almost half of all cancer patients can be candidates to receive these treatments in different settings (adjuvant, metastatic, or neoadjuvant). In addition, many more approvals are expected within the following years [1]. Metastatic melanoma is one of the tumors whose prognosis and survival have improved since ICI administration [2]. Among other benefits of ICIs in melanoma patients, one is the durable cancer control in contrast to that of other therapies like chemotherapy [3].

A decade ago, chemotherapy was the main treatment for metastatic melanoma. These chemotherapy regimens based on dacarbazine induced modest response rates ranging between 15 and 20%, being even lower for single-agent therapies [4]. However, since 2011, when the FDA approved ipilimumab, an anti-cytotoxic T lymphocyte antigen 4 (anti-CTLA4), treatment options for these patients have been growing [5]. Nowadays, the main ICI options for melanoma patients are anti-programmed death 1 (anti-PD1), like nivolumab and pembrolizumab, and anti-CTLA4 in monotherapy (ipilimumab) or, only in a metastatic setting, in combination with nivolumab (anti-PD1 plus anti-CTLA4) [6]. These drugs are monoclonal antibodies against the aforementioned checkpoint molecules.

**Figure 1:**
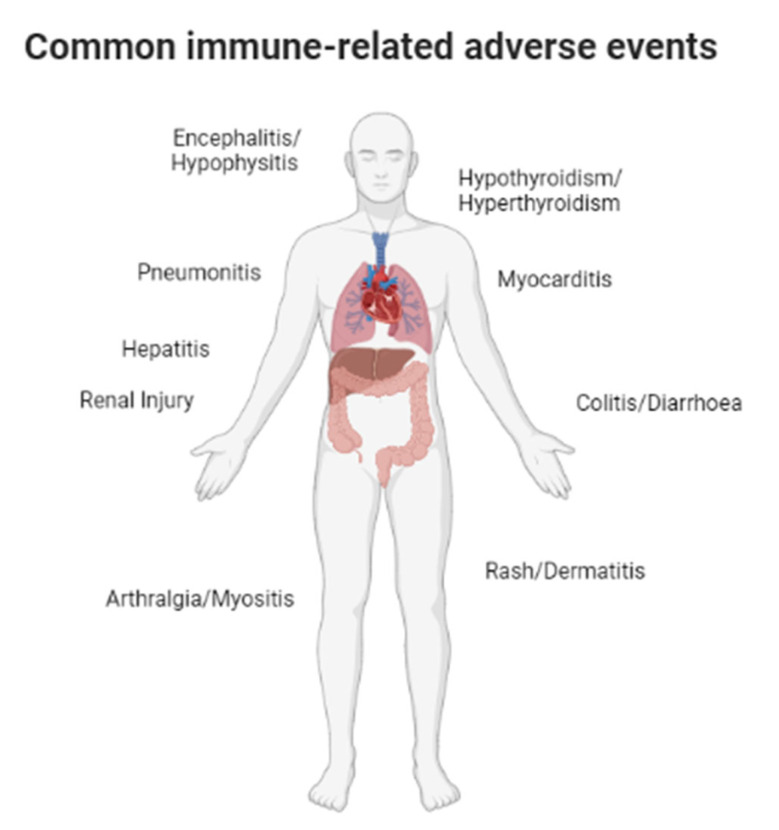
The commonest immune-related adverse events.

These new treatments brought with them a new range of toxicities due to immune system imbalance; however, the clear physiopathological mechanism related to these adverse events is unknown [7]. Immune-related adverse events (irAEs) can affect a single organ (e.g., dermatitis) or be a systemic disease affecting multiple systems [8] (Figure 1). Most common toxicities affect barrier tissues like the skin or gastrointestinal tract, among others; this is due to the regulatory role of CTLA-4 and PD-1/PDL1 in the immune cell driven in these tissues [9]. In the gastrointestinal system, colitis and hepatitis are the more frequent irAEs. In this case, colitis is the most common, affecting up to 40% of patients treated with ICI. Hepatitis is the following in this area, present in up to 30% [10]. Other less frequent irAEs are renal irAEs which are described in 2–5% of the patients treated with anti-PD1 or anti-CTLA4 [11], and cardiovascular or neurologic irAEs are rare. They affect less than 1% of patients [12,13]. Apart from these, endocrinopathies are the other common toxicities [14].

irAEs can be severe or potentially fatal when of grade 3–4, according to the CTCAE (Common Terminology Criteria for Adverse Events) [15]. Due to the unspecific symptoms associated with these irAEs, the diagnosis requires a high index of clinical intuition. Prompt suspicion and recognition of the toxicity and treatment initiation can impact directly the patients’ health and quality of life [16]. The incidence of this toxicity varies widely among patients; it usually appears within the first months of treatment but can occur at any moment during the treatment [17]. Another characteristic of these ICIs is that, currently, all have fixed doses, so modifying the dose when facing an adverse event, as with chemotherapy, is not an option.

irAEs occur more frequently in combination schedules than in monotherapy regimens [18]. For metastatic melanoma patients, the approved combination is nivolumab (anti-PD1) and ipilimumab (anti-CTLA4). The basis of this combination is their complementary roles in regulating immunity, creating a synergy between both drugs. This combination has better responses than monotherapy regimens do; however, it has a high rate of toxicity [3]. Different dosing strategies have been explored to reduce toxicity while maintaining efficacy. Checkmate 067 is a clinical trial exploring precisely this strategy. At the three-year analysis, 96% of patients had some adverse events due to treatment with the combination, 59% of them being grade 3–4. The most frequent adverse events of any grade were skin-related toxicity, while the most common grade 3–4 toxicity was gastrointestinal [19]. In the latest analysis of this clinical trial presented at ASCO 2021, no new safety issues were observed at 6.5 years [20]. On the other hand, in pembrolizumab monotherapy treatment, toxicity of any grade was 72.9%, and grade 3–4 toxicity was 10.1%, colitis and hepatitis being the main irAEs [21]. These data were maintained at the five-year analysis [22]. In nivolumab monotherapy, the rate of toxicity of any grade was 82.1%, with grade 3–4 being 16.3%. The most common irAEs were rash and hepatitis [23], without any changes at the 5–6-year analysis [20].

Between the wide range of adverse events, there are some irAEs that can be easily managed, like endocrinopathies, which usually require replacement treatment and follow-up. Nevertheless, there are other cardiologic or pneumological toxicities that are severe, the treatment more complex, and normally need to be managed by a multidisciplinary team administering immunosuppressive treatment. In fact, different medical societies are trying to construct guidelines to help clinicians recognize and treat irAEs. Among these societies is the European Society for Medical Oncology (ESMO); the aim of ESMO guidelines [24] is to provide recommendations based on evidence from the literature and clinical experience. The last version of this guideline is from October 2022. irAE management has to follow four steps: (I) diagnosis and grading according to CTCAE guides, (II) ruling out a differential diagnosis, (III) selecting the appropriate treatment strategy, and (IV) close monitoring of the irAE and its evolution.

In general, the treatment for these toxicities is immunosuppressive drugs, mainly corticosteroids. It is important to start this treatment after ruling out other diagnoses such as infections [25]. Corticosteroids are administered at 1 mg/kg/day with tapering off over at least four weeks; it is important to maintain this treatment for four weeks to avoid irAE recurrence [15]. Between 58 and 85% of irAEs are reversible with this management schedule [26], but sometimes, irAEs are steroid-refractory, so other agents are necessary, such as tumor necrosis factor (TNF)-alpha antagonists or mycophenolate mofetil (MMF). The advantage of TNF-α antagonists is its immediate therapeutic effect [27], while other immunosuppressive drugs, such as MMF or azathioprine, are effective after several weeks. However, the true situation is that prospective studies evaluating the safety and efficacy of immunosuppressant agents in irAE management are needed [24]. Another important point is ambulatory management or inpatient care, so guidelines suggest that grade ≥3 irAEs should be treated as inpatients, except when they are skin or endocrine disorders.

A key point is that, initially, the use of immunosuppressive agents was thought to be related to decreased ICI effectiveness; however, several studies reported no significant difference in response rates for those patients requiring this kind of treatment [28,29].

Related to the previous idea, the resumption or rechallenge concomitant to immunosuppressive treatment is a reality. As we will show in the following pages, there is an ample strategy adapted for clinicians to avoid irAE recurrence without affecting treatment outcomes [18].

The benefit of ICIs in melanoma patients is so high that they are shifting from later lines in metastatic disease to an adjuvant and even neoadjuvant setting [18]; therefore, this could impact the treatment range for metastatic patients. In our daily practice, it is highly likely to see patients starting ICI administration who, within a short time, develop an irAE of G3, for instance, diarrhea, so treatment must be interrupted and even corticosteroid or another immunosuppressive therapy initiated.

Clinical trials provided evidence that patients who develop a severe irAE are not allowed to resume ICI treatment. However, nowadays, new questions arise from daily practice: what should be done if G3 irAEs occur but are resolved? Should ICI treatment continue in the current manner, aware of the existence of a possible second toxicity? The number of oncologists who find themselves in this situation increases daily. To help elucidate this question, in this manuscript, we reviewed the evidence published concerning ICI resumption and rechallenge in melanoma patients.

## 2. Methods

For the literature review, a search was performed in PubMed (https://pubmed.ncbi.nlm.nih.gov/ (accessed on 1 June 2022)). The strategy used for systematic searching was the following: “immunotherapy resumption in melanoma patients”/“immunotherapy rechallenge in melanoma patients”, adding different terms according to the concept studied. The references of the articles chosen were reviewed to identify other relevant associated articles.

## 3. Description of the Evidence

Published data relating to the resumption or rechallenge of ICI treatment in melanoma patients are scarce and heterogeneous. Despite a large number of studies performed on this topic, few focused exclusively on melanoma patients (Table 1).

First of all, it is important to clarify the differences in the concepts of rechallenge, retreatment, and resumption. In general, rechallenge is defined as the reintroduction of ICI treatment after disease progression to ICI therapy [42]. Retreatment is defined by Zaremba et al. [43] as “repeated treatment with the same therapeutic class following relapse after adjuvant treatment”. Finally, resumption is defined as restarting treatment with the same drug after an interruption due to irAE that was resolved to grade 1 [44]. It is quite remarkable that, in different published articles, these definitions were quite different [44], making it difficult to standardize the meaning of these concepts. Although resumption with the same agent after full recovery is usual, class switching is another strategy. When toxicity occurs with a combination, usually, anti-CTLA4 is called off and anti-PD1 is maintained.

As mentioned before, the combination of anti-CTLA4 plus anti-PD1 is more likely to provoke irAEs, though, due to its high efficacy rate, resumption or rechallenge have been undertaken in this scenario. A publication from 2016 [30] describes the possibility of offering treatment rechallenge based on nivolumab (anti-PD1) plus ipilimumab (anti-CTLA4) after suffering grade 3 toxicity. Only three patients were reported to present neurological toxicity, hepatitis, and diarrhea as the irAEs. All were rechallenged with the same ICI combination, and two presented grade 3–4 toxicity again after rechallenging: diarrhea (increase in frequency and/or loose or watery bowel movements) [45] and colitis (a disorder characterized by inflammation of the colon) [45], with no recurrence of neurological toxicity. Rechallenging was carried out after disease progression, one and two years after the initial administration and, in one case, was accompanied by prednisolone.

Pollack et al. [31] published one of the first works on a large series of resumption patients; a retrospective study including 80 melanoma patients who experienced severe irAEs after treatment with anti-CTLA4 and anti-PD1. The most frequent irAEs were colitis and diarrhea (41% of patients), while nephritis or uveitis were observed in less than 2% of cases. The irAEs were grade 3–4 in 69% of patients and grade 2 in the rest. In 10 patients, there was more than one concurrent irAE. All patients resumed treatment with anti-PD1 in monotherapy, 39% being on immunosuppression at the time of resumption, and 21% presented persistent irAEs (grade 1–2). Recurrent G3/4 toxicity was reported in 18% of patients. A case who initially presented a grade 3 rash developed Stevens–Johnson syndrome and died. Due to these toxicities, 71% of patients with recurrent irAEs discontinued treatment. Other new toxicities appeared in 21% of patients upon resuming treatment.

Some works reviewed the safety of resumption/rechallenge in several tumor types, including melanoma. For instance, Simonaggio et al. [32] retrospectively analyzed a cohort of 93 patients with toxicity grade ≥2 after ICI treatment, 33% of whom were melanoma patients. They observed a wide variety of toxicities, such as hepatitis, pneumonitis, endocrine events, and even one cardiac event. Fifty-four percent of irAEs were grade 3–4 and the remainder were grade 2; immunosuppressive drug treatment was required in 53% of patients. Forty patients were rechallenged, and 55% of them developed irAEs again, 42.5% of these presented the same toxicity, and 12.5% had a different irAE. Of these, five patients had both a new and a recurrent irAE. After rechallenging, 62% of irAEs were grade 3–4. In another study, Allouchery et al. [33] described a cohort of 180 patients, where 43.9% were melanoma patients. Of all these patients, 48% presented grade 3–4 toxicity, and the rest were grade 2. Most patients (83.9%) were treated with anti-PD1 or anti-PDL1 monotherapy (only 10% received the anti-PD1/anti-CTLA4 combination); 88.3% of patients were on ICI rechallenge, 85% with the same drug or combination. After rechallenging, 38.9% presented at least one toxicity grade ≥2. Addtionally, Dolladille et al. [46] reported the association of 452 irAEs with ICI rechallenge. The handicap of this work was that data were retrospective and from a database (VigiBase) that contains case reports from more than one hundred countries without CTCAE grading. Thus, the rate of irAE recurrence after rechallenging (28.8%) described therein must be taken with caution.

Focusing on common toxicities, one of the main studies on ICI resumption in melanoma patients after diarrhea and colitis is that of Abu-Sbein et al. [34]. Briefly, 167 cases were analyzed, 54% being melanoma patients (the others were non-small cell lung cancer cases followed by other solid and hematologic tumors). Patients were treated with anti-PD1/PDL1 (47%), anti-CTLA4 (28%), and a combination of both (25%). Grade 3–4 diarrhea presented in 38% of patients, 33% had grade 3–4 colitis, and the rest had grade 1–2 diarrhea. Other, non-gastrointestinal toxicities were registered in 72 cases. Immunosuppression treatment with corticosteroids was required in 68% of patients. In all cases, treatment was interrupted due to toxicity and resumed in 119 patients after irAE resolution; 48 patients were rechallenged due to disease progression or relapse. When resumption occurred, no one received the anti-PD1 and anti-CTLA4 combination. Of the patients resuming treatment, 57 had toxicity recurrence of diarrhea G2 (70%) and colitis G1 (30%); 81% of these patients required corticosteroid therapy, and 12% needed another type of immunosuppressive treatment. According to the treatment received (anti-PD1/PDL1 vs. anti-CTLA4), no differences were observed in the severity of toxicity recurrence, although it appeared significantly earlier in the anti-PD1/PDL1 group. A univariate logistic regression analysis was carried out to detect factors involved in diarrhea and colitis recurrence, finding that receiving anti-CTLA4 as the initial therapy, initially requiring immunosuppressive therapy, and having high-grade (3–4) diarrhea were statistically significant factors. For irAEs colitis and diarrhea, some studies [34,47,48,49] suggested fecal calprotectin and lactoferrin as possible biomarkers to monitor treatment response and help decide the best moment for resumption/rechallenge. There is another study by Malet et al. [35] analyzing the risk of recurrence of gastrointestinal irAEs after retreatment with ICIs. There were 80 patients included, 75% of whom were melanoma patients. Of all patients, 26 received resumption/rechallenge ICI treatment, seven with anti-CTLA4 and 19 with anti-PD1. Of these retreated patients, 10 previously had grade 1–2 toxicity, and 16 had grade 3–4 irAEs. The rate of any-grade toxicity recurrence was 23%, with grade 4 being 33%. An analysis of the relationship between the drug and recurrence rate showed a higher recurrence rate when the second-line therapy was anti-CTLA-4 rather than anti-PD-1 (log-rank test: 0.11).

In this context, another common toxicity was immune-mediated hepatitis. Michael Li et al. [36] analyzed a cohort of 1913 melanoma patients treated at several centers. Of these, only 102 (5%) patients presented grade 3–4 hepatitis. Thirty-one patients were re-treated with ICI (all monotherapy), 48% developed an any-grade irAE, while six required treatment interruption due to high-grade toxicity. Hence, only 19.4% presented an important toxicity. It is remarkable that patients on rechallenge were younger, more likely to have received the anti-PD-1/anti-CTLA-4 combination, and had milder hepatotoxicity.

Another study worth highlighting is that of Cortazar et al. [37], a retrospective study of ICI-acute kidney injury (AKI) in a cohort of 138 patients, of which only 35% were melanoma patients. Most patients (92%) received anti-PD1 treatment and only a few received the anti-CTLA4 and anti-PD1 combination. After AKI resolution, 22% of patients underwent rechallenge with ICI, 87% of these received the same drug as administered previously and, of these, 39% received ICIs plus steroids. Recurrent AKI occurred in 23% of these rechallenge patients, specifically those with a shorter time between initial AKI treatment and restarting the treatment. They observed that the anti-CTLA4 and anti-PD1/PDL1 combination was an independent risk factor to develop ICI-AKI. Extrarenal irAE concomitantly with AKI occurred in 17 patients after reinitiating treatment; they presented rash, pneumonitis, and thyroid disease, among others. Some conclusions extracted from this work were that patients with extrarenal toxicity had a worse renal prognosis, and patients who did not recover from ICI-AKI had increased mortality. In parallel, Gupta et al. [38] also studied ICI-AKI in 429 patients, 24.2% of who had melanoma. A set of 121 cases were rechallenged, and 16.5% of these had recurrent ICI-AKI. Patients who developed recurrent ICI-AKI were observed to have higher mortality compared with those who did not (*p*-value = 0.02), although there were no significant differences in basal characteristics between these patients.

Evidence regarding resumption or rechallenge with other toxicities, such as neurological irAEs (ir-AEN), is less frequent because the general recommendation is not to readminister ICIs in these cases. Hence, Cuzzubbo et al. [39] reported seven cases of immune-related meningitis in melanoma patients treated with ICI. Of these, four patients received the anti-CTLA4 plus anti-PD1 combination, two received anti-PD1 monotherapy, and one patient received a combination of anti-PD1 plus BRAF (V-raf murine sarcoma viral oncogene homolog B)/MEK (mitogen-activated protein kinase enzyme) inhibitor. In general, ir-AEN were grade ≤2 except in one patient who had meningitis grade 3. ICI treatment was reintroduced in four cases, two of which received the same drug scheme again and two received a different treatment scheme. Resuming and rechallenging treatment was tolerated well, except for a patient receiving anti-PD1 in combination with a BRAF/MEK inhibitor who had interstitial lung disease grade 3.

A cardiological immune-related adverse event (ir-AEC) has the same recommendation as ir-AEN and, normally, ICI treatments are not resumed or rechallenged, which explains the scarcity of studies or clinical cases reported. There is one publication of a retrospective analysis of patients affected with immune-related myocarditis (ir-M); however, it did not include melanoma patients, but due to limited data on this toxicity, it is worth mentioning. The study [50] analyzed seven patients from 849 treated with ICIs at an Israeli center. This demonstrates the low incidence of this type of irAEs. Four of these patients (57%) were treated with anti-PD1 (three with pembrolizumab and one with nivolumab), and the rest with anti-PDL1 (atezolizumab and durvalumab). The patients were classified as myocarditis grade 1 (two patients), grade 2 (three patients), grade 3 (one patient), and grade 4 (one patient). After evaluation by a multidisciplinary committee, three patients with grade 1–3 myocarditis were selected to resume ICI treatment. After resolution of the myocarditis symptoms, due to disease progression, the patients were re-treated with ICI concomitant to corticosteroids. Only one patient had cardiological symptoms after resumption and had to stop ICI treatment. Therefore, the study concludes that resumption/rechallenge concomitant to corticosteroids should be considered in patients with low-grade ir-M together with close monitoring by an oncologist and cardiologist.

Another kind of toxicity is rheumatic. This includes arthritis, myositis, polymyalgia, and Sicca syndrome. The prevalence of these irAEs ranges from 3.3 to 6.6% [51]. Immune-related myositis has a prevalence of 1% [52]. Weill’s work [40] retrospectively analyzed data about immune-related myositis (without myocardial involvement) from several centers in France. Twenty patients were included, 15 of whom were melanoma patients. Of the melanoma patients, 10 were metastatic and five were in an adjuvant setting. The different treatments received were anti-Pd1 for 17 patients, anti-PD-L1 for one patient, and an anti-PD1 plus anti-CTLA4 combination for two patients. ICI treatment was interrupted in 18 patients, of which nine were rechallenged after toxicity resolution; six had moderate and three had severe immune-related myositis. Five patients were treated with the same drug, three were switched to another anti-PD1, and one was treated with anti-CTLA4. Of these nine rechallenged patients, six were simultaneously treated with immunosuppressive therapy in monotherapy (oral prednisone) and in combination (with methotrexate and intravenous immunoglobulins) for severe immune-related myositis. During the follow-up, only one patient experienced a new irAE, grade 3 colitis. This work supported the possibility of safe ICI rechallenge in selected cases.

Most recently, an abstract presented by Medina et al. [41] at the ASCO (American Society of Clinical Oncology) Annual Meeting in 2022 analyzed a cohort of 220 patients from a Spanish center, including 14% with melanoma. Sixty-six percent of cases were treated with anti-PD1/PDL1 in monotherapy, and 9% with a combination of anti-PD1 plus anti-CTLA4. A grade 3–4 toxicity presented in 20% of patients, but only 25% of patients experiencing toxicity required interruption of ICI treatment. After irAE resolution, 50% of patients resumed treatment, with toxicity recurrence in 82%. The most frequent recurrent irAEs were colitis (35%) and nephritis (28%).

A recent retrospective study published in December 2022 [53] examined the association between irAEs and survival in metastatic melanoma patients and included a sub-analysis of overall survival (OS) in patients hospitalized for an irAE and then resumed ICI treatment. They defined resumption after an irAE as a patient who received ≥1 cycle of ICI after withholding ICI for irAEs. There were 492 patients included in the study, the majority (75%) received anti-PD1 in monotherapy (288 patients pembrolizumab and 80 patients nivolumab), and the other 25% of patients received the anti-CTLA4 and anti-PD1 combination at different moments during disease evolution (64.6% for first-line and the rest for ≥2 lines).

The rates of toxicity grade ≥2 were 28.8% and 30.2% for nivolumab and pembrolizumab, respectively. Colitis was the most common toxicity. For the combination, 70.2% of patients had an irAE, colitis also being the main adverse event, followed by hepatitis and pneumonitis. Other important irAEs, like carditis or ir-AEN, were registered.

Of all the patients included, only 93 patients who developed an irAE resumed ICI afterwards. The article does not specify what type of toxicity the patients who underwent ICI resumption had, but they previously mentioned gastrointestinal, pneumological, cardiological, and ir-AEN as the toxicities. These are an example of the wide range of severe toxicities; however, there was no new toxicity registered after resumption. OS analysis showed a longer median OS associated with resumption compared with not resuming ICI (40.8 months vs. 61.5 months; *p* = 0.009).

## 4. Discussion

In the literature, the main evidence published regarding treatment resumption or rechallenge is retrospective, sparse, and heterogeneous, but provides useful knowledge about the safety of these strategies. Nowadays, most factors that contribute towards the use of these types of treatments again are unknown; the decision to resume or rechallenge is difficult because of the potential toxicity severity and the absence of guidelines [18].

The rechallenge or resumption strategy is based on the high effectiveness of ICI treatment because ICIs are effective for different tumor histologies and independent of driver mutations. On some occasions, it is also the only treatment available; this is the case of BRAF wild-type metastatic melanoma, where immunotherapy is the only therapeutic option. On the other hand, several high-impact publications have demonstrated a positive association between the development of irAEs and ICIs’ anti-tumor efficacy [54]. This is an important reason to support the idea of re-using this treatment despite the toxicity. In a study explained earlier [53], the patients under rechallenge had a better OS than those who were not retreated after toxicity. An Italian work [55] carried out a systematic review of studies that reported the efficacy and safety of ICI rechallenge. Forty-nine studies were included (*n* = 13,027 patients) in the analysis; most (40) were retrospective. The reason for ICI interruption was disease progression in 22 studies, toxicity in 13 studies, and both in the rest. Of all the studies, 24 were exclusively on melanoma patients, and in seven, there were various types of tumors. The treatments used the first time and for rechallenge were anti-PD1 (pembrolizumab and nivolumab), anti-PDL1 (atezolizumab), anti-CTLA4 (ipilimumab), and a combination of anti-PD1 and anti-CTLA4. In the toxicity subgroup analysis, rechallenge provided a median overall response rate of 44% and a median progression-free survival (PFS) of 13.2 months. In fact, PFS was higher in the subgroup of patients under rechallenge after toxicity compared with those who had discontinued treatment due to disease progression (11.7 vs. 3 months). OS analysis was not possible because of a lack of data. The article described that rechallenge had acceptable tolerability. In fact, patients who were rechallenged after toxicity had a lower toxicity incidence compared with those under rechallenge due to progression (46 vs. 54%). The any-grade toxicity incidence was 57.5%, and grade 3–4 incidence was 21.3%. These data are consistent with that of other studies that suggest a better response for patients who experience an irAE. A recent meta-analysis carried out by an Italian group [56] also evaluated the possible relationship between irAE incidence and improved survival (OS, PFS) and response rate. This work included 30 studies with 4323 patients, 90% of them retrospective studies with melanoma and non-small cell lung cancer patients. The patients who had an irAE had a reduced risk of death (HR = 0.49), which was associated with a reduced risk of progression (HR = 0.51).

Evaluating the efficacy of these strategies is another point to consider. This has been studied not only in melanoma but also in renal or lung tumors [57,58].

One of the first studies about these strategies in melanoma patients [59] evaluated retreatment with anti-CTLA4 in melanoma patients, with a follow-up of more than 5 years. It showed that disease control in retreatment is comparable with that observed during the first treatment.

In other studies like that of Nomura et al. [60], where pretreated melanoma patients were treated with ICI and then rechallenged, a response similar to the first ICI treatment (75%) and the rechallenge (62.5%) was seen.

Among the revised papers included in this manuscript, only four manuscripts give some efficacy data after rechallenging. In Spain et al. [30], three patients with a rechallenge had stable disease and a partial response. In Simonaggio et al. [32], 28 patients had a response (13 partial response, 15 stable disease), nine patients had disease progression, and three patients were not evaluable. In Cuzzubbo et al. [39], three patients had a partial response. Finally, in Weill et al. [40], five patients had a partial response and one had a stable disease.

A recent systematic review and meta-analysis [61] with rechallenge in different tumors concluded that there are no significant differences in efficacy between the first ICI treatment and the rechallenge. However, considering the limited data available, prospective studies are needed to confirm the efficacy of rechallenge.

In order to improve the safety of this strategy, the generation of multidisciplinary teams specialized in immune-related toxicity is highly recommended in reference centers. An example is that formed in the Gustave Roussy Hospital [32], allowing the application of their expertise to diagnosis optimization, further management, and close monitoring of patients resuming treatment after toxicity [54]. There are other strategies easily adaptable to daily practice to closely monitor patients under resumption or rechallenge after toxicity. First off is to train patients to recognize signs or symptoms and consult the emergency department. A specific anamnesis about symptoms of previous toxicity is necessary. As, in the general follow-up, a blood test is required prior to each cycle, in which renal and liver function as well as hematocrit, white blood cells, and platelets must be evaluated; TSH/T4 are other biomarkers accessible in daily practice, and other previously mentioned inflammatory markers such as calprotectin might also help.

Reviewing the previous articles, some toxicities have greater possibilities of recurrence after resumption or rechallenge. Pollack et al. [31] reported hepatitis, pancreatitis, pneumonitis, and nephritis as likely to recur, while colitis did not present a high recurrence rate. Contrarily, in other studies [30,34], colitis was more likely to recur, reaching statistical significance. After other irAEs, such as neurological (irAEN), it was not usual to perform resumption or rechallenge, although some studies [30,31,39] presented evidence that ir-AEN do not usually recur and it could be safe to resume or rechallenge in specific situations. The same scenario was observed for initial endocrine irAEs; according to Allouchery’s study [44], endocrinopathies were less likely to recur (*p* = 0.003). Thus, these pieces of evidence suggest ideas that the type of toxicity has an impact on the likelihood of recurrence and that the rate of recurrent irAEs changes according to the system involved in the initial irAE [44].

In daily practice, it is easy to identify some clinical characteristics that could exert an influence on increasing the risk of irAE appearance, such as an ECOG (Eastern Cooperative Oncology Group) ≥2 or a previous history of immune disease. Unfortunately, in melanoma patients, it was quite difficult to derive conclusions on this topic because there were no specific publications. From the aforementioned retrospective works, we could extract some statements: according to Abu-Sbeih et al. [34], patients who resumed anti-CTLA4 treatment presented toxicity recurrence earlier than those with anti-PD1/PDL1; Chennamadhavuni et al. [62] reviewed risk factors for developing irAEs in immune-naive patients. The risk factors for developing an irAE were grouped into three categories:

(a) Patient-specific. This group includes demographic and social history data. Age, race, or gender could be involved in the risk of suffering an irAE. Obviously, other patient characteristics like ECOG or physical status (sarcopenia, level of muscle mass) could also affect it. Medical history is important too; for instance, a previous history of autoimmune disease increased the irAE risk. Finally, some studies suggested that antibiotics or influenza vaccinations, for example, could be involved in immune toxicity.
(b) Tumor-specific. There are characteristics such as disease burden or higher tumor mutational burden (TMB) that affect disease evolution and treatment evolution. It is worth highlighting that some tumors are more related to some specific toxicities. For instance, melanoma is more related to diarrhea, colitis, and skin toxicities than other tumors like non-small cell lung cancer.
(c) Agent-specific. As we mentioned previously, the incidence and type of toxicity differ according to the use of anti-PD1 or anti-CTLA4 in monotherapy or in combination.

This information could be extrapolated to melanoma patients; however, there was great variability between works, thus the evidence must be interpreted carefully. Another very useful aspect would be the discovery of specific biomarkers to help identify patients more prone to presenting toxicities and even detect early toxicities in patients who already had one. Currently, some research teams are working on generating preclinical models to better understand how or why some toxicities appear with ICI treatment [63].

The work of Chennamadhavuni et al. [62] suggested several types of biomarkers that could be related to irAEs; these include circulating blood cell counts, cytokines, autoantibodies, serum proteins, HLA genotypes, microRNA, gene profiling, and intestinal microbiota. Some examples of these biomarkers are described below. According to circulating blood cell counts, a study [64] observed that a rise in white blood cell count (59%) and a drop in relative lymphocytic count (32%) from the baseline level is related to lung or gastrointestinal irAEs in melanoma patients treated with anti-PD1 (nivolumab). Regarding cytokines, there is a study [65] that evaluated some functionally selected cytokines and chemokines prior to treatment with ipilimumab (anti-CTLA4) and six weeks afterwards. This study showed that IL-17 (interleukin 17, a pro-inflammatory molecule produced by T-helper 17 cells related to auto-immune diseases [66]) correlated significantly with the development of grade 3 diarrhea and colitis when it increased between baseline and six weeks. Pistillo et al. [67] showed that a high pretreatment level of soluble CTLA4 antibody (>200 pg/mL) correlated with gastrointestinal irAEs in melanoma patients treated with ipilimumab. The intestinal microbiota is a popular concept nowadays; it is well known that the composition of the intestinal microbiota can affect the development of general inflammatory disorders and, more specifically, gastrointestinal inflammatory disorders. A study of 34 melanoma patients treated with anti-CTLA4 (ipilimumab) with some specific microbes from the Bacteroidetes phylum were associated with a reduced risk of colitis [68].

These findings could be crucial to identify and evaluate specific subgroups of patients who may benefit from resumption/rechallenge strategies.

## 5. Conclusions

The main limitation of this work comes from the limited number of publications regarding the main topic in melanoma patients and also the retrospective nature of the evidence available in the literature. It is hard to construct concrete conclusions about the best approach with the available studies because there are many factors involved in resumption/rechallenge strategies like primary tumor, involved organ, and the ICI drug used. The application of resumption or rechallenge as therapeutic strategies is possible, but each patient should be evaluated by a multidisciplinary team to closely monitor and assess the risk–benefit ratio before initiating treatment. Finally, prospective studies and biomarkers are needed to guide clinicians in practical decisions.

## Figures and Tables

**Table 1:** Main studies published regarding ICI resumption/rechallenge after toxicity in melanoma patients.

	Number of Patients (*n*)	Melanoma Patients (*n*)	Number of Patients on Resumption/Rechallenge *	% irAE Recurrence, Any Grade	% irAE Recurrence, Grade 3–4
Spain et al. [30]	3	3	3	100	66
Pollack et al. [31]	80	80	80	50	18
Simonaggio et al. [32]	93	31	40	100	62
Allouchery et al. [33]	180	79	159	NA	38.9
Abu-Sbeih et al. [34]	167	90	167	34	82
Malet et al. [35]	80	60	26	23	33
Li et al. [36]	102	102	31	48	19.4
Cortazar et al. [37]	138	49	31	23	NA
Gupta et al. [38]	429	104	121	16.5	40
Cuzzubbo et al. [39]	7	7	4	NA	14.2
Weill et al. [40]	20	15	9	NA	1.1
Medina et al. [41]	220	32	17	NA	82

NA: not available. * In Simonaggio et al., there were 11 melanoma patients in the rechallenge group; 6 had irAE recurrence, but the grade was not specified. In Allouchery et al., all melanoma patients included were rechallenged (79 patients); 33 patients had irAE recurrence without specifying the grade. In Gupta et al., there were 39 melanoma patients rechallenged. In Weill et al., there were 8 melanoma patients included in the rechallenge group.

## Data Availability

No new data were created or analyzed in this study. Data sharing is not applicable to this article.
